# Multi-scale simulations of two dimensional material based devices: the NanoTCAD ViDES suite

**DOI:** 10.1007/s10825-023-02048-2

**Published:** 2023-06-05

**Authors:** Damiano Marian, Enrique G. Marin, Marta Perucchini, Giuseppe Iannaccone, Gianluca Fiori

**Affiliations:** 1 Dipartimento di Ingegneria dell’Informazione, Università di Pisa, Via G. Caruso 16, 16122 Pisa, Italy; 2 Departmento de Electrónica, Universidad de Granada, Avenida Fuente Nueva s/n, 18071 Granada, Spain

**Keywords:** NanoTCAD ViDES, Multi-scale approaches, Non-equilibrium Green functions, Open-source, Nanoelectronics, Elastic transport, dissipative transport, Drift-diffusion, Printed-devices

## Abstract

NanoTCAD ViDES (Versatile DEvice Simulator) is an open-source suite of computing codes aimed at assessing the operation and the performance of nanoelectronic devices. It has served the computational nanoelectronic community for almost two decades and it is freely available to researchers around the world in its website (http://vides.nanotcad.com), being employed in hundreds of works by many electronic device simulation groups worldwide. We revise the code structure and its main modules and we present the new features directed towards (i) multi-scale approaches exploiting ab-initio electron-structure calculations, aiming at the exploitation of new physics in electronic devices, (ii) the inclusion of arbitrary heterostructures of layered materials to devise original device architectures and operation, and (iii) the exploration of novel low-cost, green technologies in the mesoscopic scale, as, e.g. printed electronics.

## Introduction

NanoTCAD ViDES is an open-source software for the simulation of nanoelectronics devices. Following the open-source community spirit, it has become, over the last two decades, a constructive alternative to proprietary commercial suites, being exploited in several hundreds of theoretical studies and becoming a valuable tool for a large number of users in the computational nanoelectronic community. Like other open-source codes, it has sought to contribute to prime scientific collaboration over competition and emphasize edge science accessibility and reproducibility. In its first version, NanoTCAD ViDES included the self-consistent solution of the Poisson and Schrödinger equations, in any dimension, using the non-equilibrium Green’s function (NEGF) formalism, and employing a tight-binding level description of the material in the electronic device under study. The code consisted (and it keeps in this way) of several interconnected modules, exploiting the versatility of Python scripting, for a high-level and easy modification by any user, interfaced with C- and Fortran-optimized subroutines. This early version of NanoTCAD ViDES already offered the possibility to simulate arbitrary materials, architectures, and geometries. It was released coincidentally to the discovery and subsequent surge in two-dimensional materials (2DMs) research, and has thus been associated to the study of 2DMs based electronic devices including, in the investigations in graphene nanoribbons [1], carbon nanotubes [2], or bi-layer graphene field effect transistors [3], but also low-dimensional structures of conventional materials as, e.g., silicon nanowires [4].

Over the years, NanoTCAD ViDES has been enriched with several features that have raised its functionalities both from the material level as well as from the architectural perspective. First, it has been interfaced with Wannier90 suite [5], thus enabling a detailed description of the material band-structure. In particular, the Wannier tight-binding like Hamiltonian, expressed in terms of maximally localized Wannier functions (MLWF) has allowed the connection of NanoTCAD ViDES with electronic-structure codes based on Density Functional Theory (DFT) such as Quantum ESPRESSO [6], SIESTA [7], VASP [8], etc. This particular upgrade has allowed the code to exploit all the accuracy of ab-initio DFT calculations, making it fully multiscale. Second, the code has been raised with the ability to simulate lateral [9] and vertical [10] heterostructures (HS), with full ab-initio accuracy. To this purpose, the Wannier Hamiltonian of the HS is carefully split (exploiting Wannier orbital projections) into its constituent single-layer 2DMs [10–12]. This is the most precise multi-scale approach to the modeling of van-der-Waals and lateral heterostructures as it preserves all coupling information between the layers from an ab-initio level. The improvements in the material level description have come hand-in-hand with an exponential increase of the computational burden, which is eased by the code parallelization, allowing the user to simulate complex structures exploiting high-performance computing resources. Finally, more recently the transport modules have been extended to consider dissipative transport in homogeneous media and in layered heterostructures, by a combination of the semi-classical drift-diffusion theory with ab-initio calculations [13].

In this paper, we revise the most important as well as novel contributions to the NanoTCAD ViDES code. The article is organized as follows: in Sect. 2, we introduce a general perspective on the code operation and present its core elements and structure. In Sect. 3, we present how the code deal with elastic transport, starting with a revision of the main ingredients for the definition of a tight-binding Hamiltonian in the NanoTCAD ViDES format (from the simplest example up to its connection with Wannier Hamiltonians and the formation of heterostructures) followed by a short recall of the main equations defining the transport calculation. In Sect. 4 dissipative transport is then analyzed: we briefly recollect semi-classical drift-diffusion theory, followed by a discussion about the application of semi-classical theory to 2DM layered structures. In Sect. 5, we revise some meaningful examples of the application of NanoTCAD ViDES to the study of nanoelectronic devices and in Sect. 6 we draw the conclusions.

**Fig. 1:**
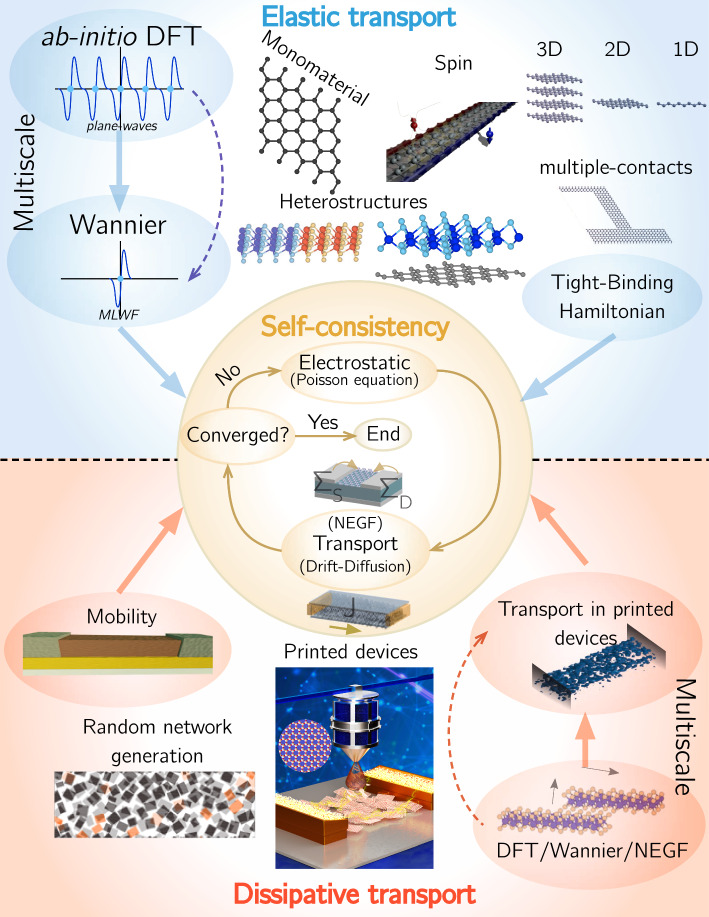
Schematic depiction of the NanoTCAD ViDES software. (Central panel) Core of the software with the self-consistent solution of the electrostatis, through the solution of the Poisson equation, coupled with the transport, that can be the solution of the time-independent Schrödinger equation, through non-equilibrium Green’s function formalism, for quantum elastic transport, or the drift-diffusion and continuity equations for the semi-classical dissipative transport. (Top panel) Pictorial depiction of the different structures that can be simulated in the elastic transport regime, following either a multiscale approach or a direct definition of the Hamiltonian through a tight binding model. (Bottom panel) Pictorial depiction of the structures that can be simulated in the dissipative transport regime following either a direct definition of the mobility and effective mass or through a multiscale approach.

## Code description

NanoTCAD ViDES enables the self-consistent solution of the electrostatics (through the Poisson equation) and the electronic transport either in the quantum elastic regime (so coupled with the time-independent Schroedinger equation, through the non-equilibrium Green’s function (NEGF) formalism) or in the semi-classical dissipative regime (i.e. coupled with the drift-diffusion and continuity equations), in a nanoscopic or mesoscopic device of any dimension (3D, 2D, and 1D). In Fig. 1, we report a visual representation of the code main features. The top chart of Fig. 1 summarizes elastic transport attributes, highlighting the two main approaches for the materials properties definition: (i) the *direct* definition of the Hamiltonian by the user in the tight-binding formalism, and (ii) the *multiscale* procedure, that exploits other codes that are interfaced with NanoTCAD ViDES thanks to Wannier90. This scheme enables the simulation of very different nanoelectronic devices, from novel 2DM-based transistors [14], to lateral heterostructures [9, 15] or vertical heterostructures [10] of 2DMs, including lower dimensional structures of different 2D materials such as choreographed graphene [16], or stanene, with the possibility of studying spin transport [17, 18], nanowires [4] and devices with many contacts [19].

The bottom chart of Fig. 1 reports the semi-classical and dissipative transport counterpart. Again, we highlight the two main approximations to the definition of the material characteristics distinguishing: (i) homogeneous channel materials, where one has to define the mobility and the effective mass of the material, and (ii) heterostructures of layered materials where the layer in-plane semi-classical diffusion of carriers is combined with the multi-scale description of the inter-flake vertical conductance. This latter feature of the code is suited for the simulation of printed devices [13, 20], by the generation of a random structure of flakes.

Once the material properties are defined, either in the quantum elastic or semi-classical dissipative regimes, the core of NanoTCAD ViDES code (schematically depicted in Fig. 1 as a central circle) is dedicated to the self-consistent solution of the transport (NEGF in the elastic regime or drift-diffusion *plus* continuity equations in the dissipative regime) with the electrostatic (Poisson equation).

## Quantum elastic transport

The first core of the NanoTCAD ViDES code, which has been developed and extended over the years, was dedicated to the solution of elastic transport, aiming at simulating devices in the *so-called* ballistic regime, i.e., devices where typical lengths are less or equal to the electron mean free path of the investigated material. As schematically sketched in the introductory section, the main ingredients for the simulation of a device in the elastic regime are: (i) a Hamiltonian in the tight-binding (TB) formalism and (ii) the self-consistent solution of the transport with the NEGF formalism coupled with the electrostatic solution of the Poisson equation. In the following, we go into more detail about the formal aspects of the code.

### Tight-binding Hamiltonian

**Fig. 2:**
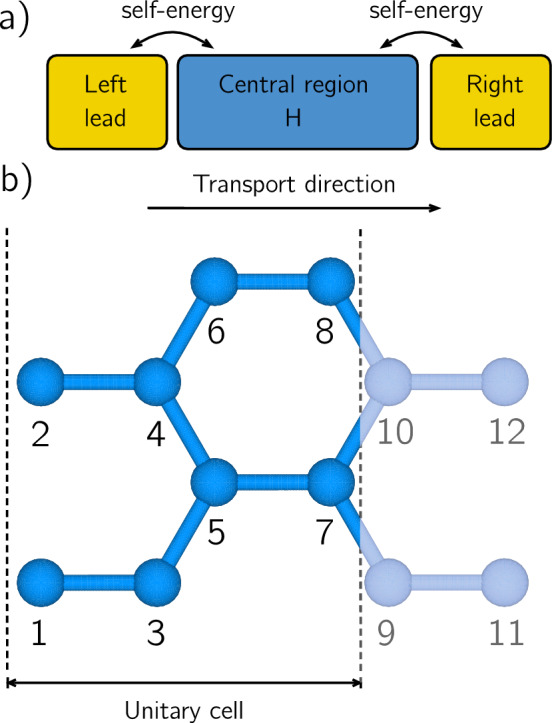
**a** Schematic depiction of the central region where the Hamiltonian (H) of the system is defined, with the left and right leads connected through the self-energies. **b** The elementary cell of an armchair nanoribbon, whose Hamiltonian is reported in Eq. (2). Transport direction is also indicated.

The elastic transport modules of the code are fed by a Hamiltonian, with a specific format, that completely characterizes the electronic structure and properties of the central (commonly called the *channel*) region of the device under study. This region is automatically extended by the code with semi-infinite leads that are attached at its right and left ends, and whose electronic properties are defined (as will be explained later) by the Hamiltonian sub-matrices corresponding to the first and last unit cells of the central region as depicted in Fig. 2a, through the self-energies. The tight-binding Hamiltonian of the central region must be written in the ViDES Hamiltonian Format (VHF) as follows:

1 $$\begin{aligned} {\mathcal {H}} = \left( \begin{array}{cccccc} n_{o} &{} 0 &{} 0 &{} 0 &{}... &{} 0 \\ 1 &{} 1 &{} h(11)_{11} &{} h(11)_{12} &{}... &{} h(11)_{n_{o}n_{o}} \\ 1 &{} 2 &{} h(12)_{11} &{} h(12)_{12} &{}... &{} h(12)_{n_{o}n_{o}} \\ 1 &{} 3 &{} h(13)_{11} &{} h(13)_{12} &{}... &{} h(13)_{n_{o}n_{o}} \\ ... \\ ... \\ i &{} j &{} h(ij)_{11} &{} h(ij)_{12} &{}... &{} h(ij)_{n_{o}n_{o}} \\ ... \\ ... \\ \end{array} \right) \end{aligned}$$

where $n_{o}$ is the number of orbitals for each atom and the matrix element $h(ij)_{nm}$ represents the hopping element *nm* between atoms *i* and *j*.

In order to illustrate the VHF, let’s consider a simple example: a graphene nanoribbon as the one depicted in Fig. 2b, where carbon atoms have been ordered as in the picture. The Hamiltonian is built in the single $p_z$ TB set in the real space, with only one orbital per atom, considering an on-site energy equal to 1 eV, a hopping parameter equal to $t=-2.7$ eV between $C-C$ atoms and $t=-3.0$ eV for the outer dimmer lines, to take into account edge relaxation. The resulting Hamiltonian in the VHF is written as:

2 $$\begin{aligned} {\mathcal {H}} = \left( \begin{array}{ccc} 1 &{} 0 &{} 0 \\ 1 &{} 1 &{} 1 \\ 2 &{} 2 &{} 1 \\ 3 &{} 3 &{} 1 \\ 4 &{} 4 &{} 1 \\ 5 &{} 5 &{} 1 \\ 6 &{} 6 &{} 1 \\ 7 &{} 7 &{} 1 \\ 8 &{} 8 &{} 1 \\ 1 &{} 3 &{} -3.0\\ 2 &{} 4 &{} -2.7\\ 3 &{} 5 &{} -2.7\\ 4 &{} 5 &{} -2.7\\ 4 &{} 6 &{} -2.7\\ 5 &{} 7 &{} -2.7\\ 6 &{} 8 &{} -3.0\\ 7 &{} 9 &{} -2.7\\ 7 &{} 10 &{} -2.7\\ 8 &{} 10 &{} -2.7\\ ... &{}... &{}...\\ \end{array} \right) \end{aligned}$$

where, for the sake of brevity, we have explicitly written down only the elementary cell. It is worth noting that in the VHF the exact order of the elements in the Hamiltonian is not relevant. For example, in Eq. (2) we have first indicated the on-site energies (1 eV in this case) and then the hopping parameters ($-2.7$ eV and $-3.0$ eV), but any other order is compatible with the code. NanoTCAD ViDES already incorporates specific functions for creating TB Hamiltonians for graphene nanoribbons (GNRs), carbon nanotubes (CNTs), silicon nanowires (NWs), etc., which only need as input the geometrical features. For example, in the case of the GNR it is enough to specify the number of dimer lines and the number of slices. An example of a more complex *decorated* graphene nanoribbons is reported in Ref. [16] which is also included in Sect. 5 of the present article.

### Multiscale approach

Despite the variety of possible systems that can be built and investigated with simple TB Hamiltonian constructions, the most powerful approach for simulating devices in NanoTCAD ViDES is enabled by its connection with DFT codes through Wannier90, i.e., defining the Hamiltonian in the so-called multiscale approach.

**Fig. 3:**
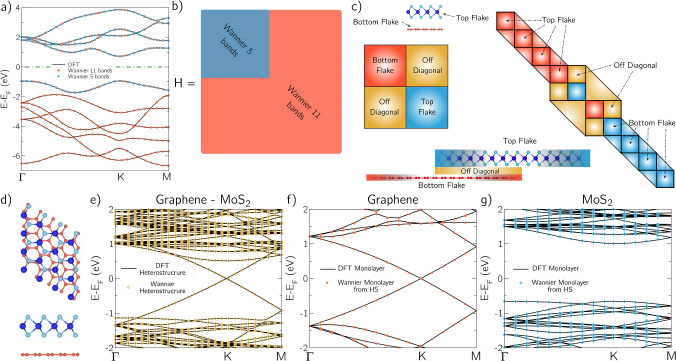
**a** Comparison of DFT bands (black lines) with the Wannier90 bands (red and blue points) for monolayer MoS$_2$ along the $\Gamma$-K-M k-path and **b** Schematic depiction of the Hamiltonian size for 11 and 5 bands in MoS$_2$ **c** Lower part: schematic depiction of a vertical heterostructure consisting of two flakes of graphene and MoS2 partially overlapped in the central region. Upper left part: schematic depiction of the bilayer Hamiltonian, with the three different blocks representing (i) the top layer (bottom right), (ii) the bottom layer (top left) and (iii) the off-diagonal elements that connect the two layers. Upper right part: schematic depiction of the construction of the complete Hamiltonian with arbitrary single flake and overlapping regions. The triangles represent the connections between adjacent cells along the structure. **d** Top and lateral view of the graphene-MoS2 supercell consisting of 5$\times$5 graphene cells and 4$\times$4 MoS_2_ cells. **e** DFT (black solid lines) and Wannier (symbols) band structures along the $\Gamma$-K-M k-path of the Graphene-MoS$_2$ heterostructure. Comparison of monolayer Graphene **f** and MoS$_2$ **g** band structure along the $\Gamma$-K-M k-path: in red **f** and blue **g** circles, the ones obtained with the projection method explained in the manuscript applied to the bilayer graphene-MoS$_2$ heterostructure, in black lines the isolated monolayer band structure obtained from DFT.

In more detail, the multiscale approach implemented in NanoTCAD ViDES works as follows. First, the electronic structure of the material, or the heterostructure of materials, is computed by means of DFT, for example, exploiting the open-source code Quantum Espresso (QE) [6]. Second, Wannier90 code [5] is employed to transform the plane-wave-based Hamiltonian given by QE into a basis of the maximally localized Wannier functions (MLWF). Third, the Wannier90 Hamiltonian, which is already in a TB-like form, is translated into the VHF format as in Eq. (1), by a proper module of NanoTCAD ViDES. It is worth recalling that Wannier90 is interfaced with many ab-initio codes, such as VASP [8], Abinit [21], CASTEP [22], Wien2K [23], FLEUR [24], Siesta [7], so any of these codes can be used to determine the electronic structure in the first step of the multiscale procedure. The Wannier90 Hamiltonian retains, in this way, all the accuracy of the ab-initio calculations, enabling the simulation of the device while preserving the precise material properties. As an example, we report in Fig. 3a the comparison between the band structure of monolayer MoS$_2$ obtained from the QE suite [6] and Wannier90 [5]: we can observe an excellent agreement between both band structures.

There are two attractive features of the Wannier90 Hamiltonian that deserve special mention. First, Wannier90 has the possibility to select a restricted number of bands in the desired energy range (for transport it usually corresponds to the energies around the Fermi level), through proper orbital projections and/or energy disentanglement ( [9, 25]). For example, Fig. 3a shows the wannieriation of monolayer MoS$_2$, with a different number of bands: (i) 11 bands (or equivalently 11 Wannier centers), which corresponds to the full valence and conduction band considering all Mo *d*-orbitals and S *p*-orbitals; (ii) 5 Wannier bands, considering only the Mo *d*-orbitals, which capture the entire conduction band and the first band in the valence region. Both cases are in excellent agreement with the DFT results, but the latter give the opportunity to substantially reduce the computational burden. Figure 3b schematically depicts the Hamiltonian size for the different number of bands or equivalently for a different number of Wannier centers. The numerical and time cost of the elastic transport calculations is significantly reduced with the Hamiltonian size (in this case reducing the complexity by a factor of $\approx 10$).

The second important characteristic enabled by Wannier90 is that by a proper projection of the bands of a layered heterostructure, it is possible to extract the Hamiltonians of its constituent layers. This enables the construction of Hamiltonians, and subsequently the study of electronic devices, where regions of different numbers and types of 2DMs are seamlessly combined, preserving all the information about the electronic coupling between layers. This is, especially attractive for computing interlayer transport, which is extremely expensive through DFT methods, and hard to properly capture from pseudo-empiric tight-binding Hamiltonians.

In order to better exemplify this feature, Fig. 3c -bottom, depicts a simple layered heterostructure with one graphene monolayer and one MoS$_2$ monolayer, that are partially overlapped. From a structural and electronic point of view, there are three clearly distinguishable spatial regions: an area with only monolayer graphene, an area corresponding specifically to the heterostructure of graphene and MoS$_2$, and an area with monolayer MoS$_2$. The starting point for the construction of the overall Hamiltonian, is a DFT calculation of the isolated bilayer heterostructure (Fig. 3d). Then, the bilayer, Fig. 3e, is wannierized by carefully choosing the initial orbital projections (that will vary depending upon the heterostructure and materials composition). For example, in the graphene-MoS$_2$ bilayer, as described in detail in Ref. [10], one should select the 3 *p*-orbitals and 1 *sp*$^{2}$-orbital for each C atom; and 5 *d*-orbitals for each Mo atom and 3 *p*-orbitals and 1 *s*-orbital for each S atom as initial projections. In general, the projections can be inferred by studying the projected density of states (PDOS) of the DFT heterostructure. If they are properly selected, and the Wannier centers are properly localized around the atoms positions, the resulting Hamiltonian has the form illustrated in Fig. 3c-upper left and Eq. (3):

3 $$\begin{aligned} {\mathcal {H}} = \begin{pmatrix} {\mathcal {H}}_{\text {BF}} &{} {\mathcal {H}}_{\text {OD}}\\ {\mathcal {H}}^*_{\text {OD}} &{} {\mathcal {H}}_{\text {TF}} \end{pmatrix}. \end{aligned}$$

It is possible to distinguish three distinct blocks in the Hamiltonian of the bilayer: (i) the upper-left block, ${\mathcal {H}}_{\text {BF}}$, that can be identified with the electronic states of the bottom layer, e.g. the graphene, (ii) the lower-right block, ${\mathcal {H}}_{\text {TF}}$, that can be identified with the electronic states of the top layer, e.g. the MoS$_2$, and (iii) the upper-right, ${\mathcal {H}}_{\text {OD}}$, and the bottom-left, ${\mathcal {H}}^*_{\text {OD}}$, off-diagonal blocks (transpose conjugates of each other), that can be identified with the connections between the electronic states of the two flakes. In this way, the bilayer Hamiltonian encodes all the information needed to compute the transport in the whole layered heterostructure of these two partially overlapped flakes. This is because the sub-matrices of the bottom and top flakes, i.e. ${\mathcal {H}}_{\text {BF}}$ and ${\mathcal {H}}_{\text {TF}}$, are clearly distinguishable and provide the bands of the isolated monolayers of graphene and MoS$_2$, as shown in Fig. 3f–g, where the DFT electronic band-structures of monolayer graphene and MoS$_2$ are compared to the ones obtained from the wannierized heterostructure Hamiltonian, i.e., ${\mathcal {H}}_{\text {BF}}$ and ${\mathcal {H}}_{\text {TF}}$. Owing these ingredients, it is possible to construct the Hamiltonian of the entire heterostructure device (comprising the 3 regions in the example) by selecting arbitrary sections of monolayer and overlap regions, and replicating the ${\mathcal {H}}_{\text {BF}}$ and ${\mathcal {H}}_{\text {TF}}$ blocks and ${\mathcal {H}}_{\text {OD}}$, as schematically reported in Fig. 3c - upper right panel.

### Self-consistent solution in the elastic transport regime

Once the Hamiltonian of the whole device is obtained (and translated into the VHF format) the transport problem is solved through the solution of Green’s function in the elastic regime [26]:

4 $$\begin{aligned} G(E) = \left[ EI - H - \Sigma _{\text {S}} - \Sigma _{\text {D}} \right] ^{-1} \end{aligned}$$

where *E* is the energy, *I* is the identity matrix (because the TB or MLWF Hamiltonian is expressed in an orthorhombic basis set), and $\Sigma _{\text {S}}$ and $\Sigma _{\text {D}}$ are the self-energies of the source and the drain respectively. In more detail, NanoTCAD ViDES implements the recursive Green’s function method [27] with a complexity of $O(N_{\text {C}}N_{\text {wc}}^3)$, where $N_{\text {C}}$ is the number of slices in the transport direction and $N_{\text {wc}}$ is the number of Wannier centers (or the number of atoms in the simplest nearest-neighbor TB Hamiltonians), while for the self-energies, the closed form derived in [28] is adopted. Once *G*(*E*) is computed, one can calculate all the relevant quantities, such as the free charge concentration in the correspondence of each Wannier center/atom (which is then included in a self-consistent scheme with Poisson equation, see Fig. 1 central panel), the transmission coefficient, *T*(*E*), and eventually the current flowing using Landauer’s formalism:

5 $$\begin{aligned} I = \frac{2q}{h} \int _{-\infty }^{+\infty } T(E) \left[ f(E-E_{\text {FS}}) - f(E-E_{\text {FD}}) \right] dE \end{aligned}$$

where *q* is the electron charge, *h* is the Planck’s constant, and $E_{\text {FS/FD}}$ are the Fermi level at the source/drain.

## Semi-classical dissipative transport

NanoTCAD ViDES also includes modules able to treat semi-classical dissipative transport, i.e. aiming at the simulation of electronic devices at the mesoscopic scale, where the device length is many times longer than the electron mean free-path of the investigated material. In particular, the dissipative transport (comprising drift-diffusion and continuity equations) is solved. self-consistently with the electrostatics (Poisson equation) either in conventional homogeneous media or layered heterostructures of 2DMs. In the latter case, a combination of the semi-classical drift-diffusion theory and ab-initio calculations is employed in a multi-scale fashion [13]. In the following, we go into more detail about the formal aspects of this part of the code.

### Drift-diffusion for homogeneous materials

The semi-classical theory for dissipative transport in homogeneous media is governed by the continuity equation in stationary conditions (i.e. $\nabla J = 0$). In NanoTCAD ViDES the current density, *J*, is defined using the quasi-Fermi level approach [29], which has been demonstrated to be numerically more stable and accurate than charge-based implementations [30]:

6 $$\begin{aligned} J(\textbf{r}) = q \mu _n(\textbf{r}) n(\textbf{r}) \nabla E_{f_n}(\textbf{r}) + \mu _p(\textbf{r}) p(\textbf{r})\nabla E_{f_p}(\textbf{r}) \end{aligned}$$

where $\mu _n$ and $\mu _n$ are position-dependent anisotropic electron and hole mobility, *n* and *p* are the carrier densities of electrons and holes respectively, and $E_{f_n}$ and $E_{f_p}$ are  the spatial-dependent quasi-Fermi levels. The carrier densities are determined by the specific Fermi-Dirac integral, which depends on the channel material dispersion relationship and the dimension (1D, 2D, 3D) of the channel region of the device under study.

The general flow chart of the algorithm is as follows: Poisson and continuity equations are self-consistently solved to determine respectively the potential (*V*) and the $E_{f_{n/p}}$ distributions. The carrier densities, which depend on both *V* and $E_{f_{n/p}}$ values, are updated after every solution of the electrostatic and the transport, and used in the next iteration. In more detail, the iterative process starts with the initial guess of the potential in equilibrium (setting $E_{f_{n/p}}=0$), under proper Dirichlet boundary conditions for the contacts. Then, the charge densities in equilibrium are evaluated and introduced in the continuity equation, which is solved giving $E_{f_{n/p}}$, which is used to update the charge and continue the iterative process (using a conjugate gradient iterative method) until convergence is reached, and the current density is determined.

### Simulation of printed networks of 2DMs

The dissipative transport module goes far from conventional drift-diffusion theory when dealing with heterostructures of layered materials on the mesoscopic scale, as for example the one formed in printed devices. In this case, while the equations governing the electrostatics and transport do not change, but the transport between layers is treated more accurately by employing a multi-scale scheme that exploits ab-initio calculations between layers, similar to the procedure exposed in Sect. 3.2 and described in detail in Refs. [13, 20]. This is especially relevant because, in layered heterostructures of 2DMs, the transport along the layer (in-layer) and between layers (inter-layer) significantly differs, thus producing a transport anisotropy that can be treated by means of an anisotropic mobility.

**Fig. 4:**
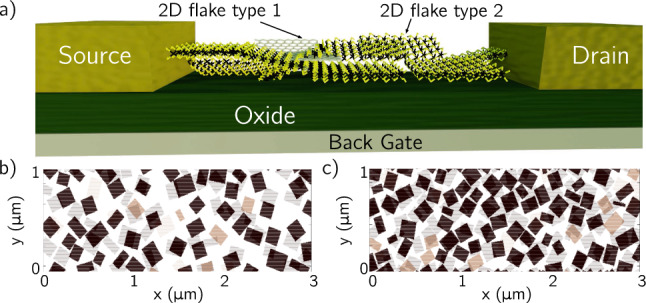
**a** Schematic depiction of a 2DM-ink based printed device. **b, c** The channel is a network of two different types of 2DM flakes (90$\%$ of material 1 (grey) and 10$\%$ of material 2 (orange)) with random sizes and orientations. Network structure sections in two adjacent planes (indicated with different level of transparency) perpendicular to the vertical direction, for FF = 0.4 **b** and FF = 0.7 **c**. In these networks, rectangular flakes with an average lateral size of 150 nm and random orientation are considered

In particular, NanoTCAD ViDES implements the possibility of simulating printed devices based on 2DMs, as the one schematically depicted in Fig. 4a. In this particular structure, there is a network of flakes, with dimensions of the order of $\mu$m’s, i.e. the size of the printed patterns, which is composed of flakes of the order of hundreds of nm’s (the size of the flakes dissolved in the ink [31]).

In order to model electronic devices based on this technology it is necessary to consider the random nature of the distribution and interconnection of flakes in printed networks. To this purpose, the code includes a random generator module based on a Monte Carlo approach that builds a network with a desired density of flakes, defining every individual flake in the network with a random size and orientation within a 3D grid.

In more detail, the Monte Carlo module, defines first the shape of the flakes, which can be a circle or a parallelogram, and their thickness. Second, it generates the flake random size (i.e radius, or lateral dimension) and orientation using a normal distribution of desired mean and variance values for both. Third, the density of flakes in the network is set by defining the filling factor (FF), i.e., the ratio of the volume occupied by the flakes with respect to the total volume of the printed channel. Finally, an origin point is randomly generated for each flake in which the flake is placed with the corresponding dimension, orientation, and size. Figure 4b-c, shows a couple of networks generated by the algorithm. In particular, two adjacent planes perpendicular to the vertical (z) direction are plotted with FF = 0.4 and FF = 0.7.

A grid mask is created by the module, assigning at each point the different materials properties and in particular, the spatial-dependent anisotropic mobility, treated in the way described in Refs. [13, 20], to be used in the solution of Eq. (6).

## Examples of simulated devices through NanoTCAD ViDES

**Fig. 5:**
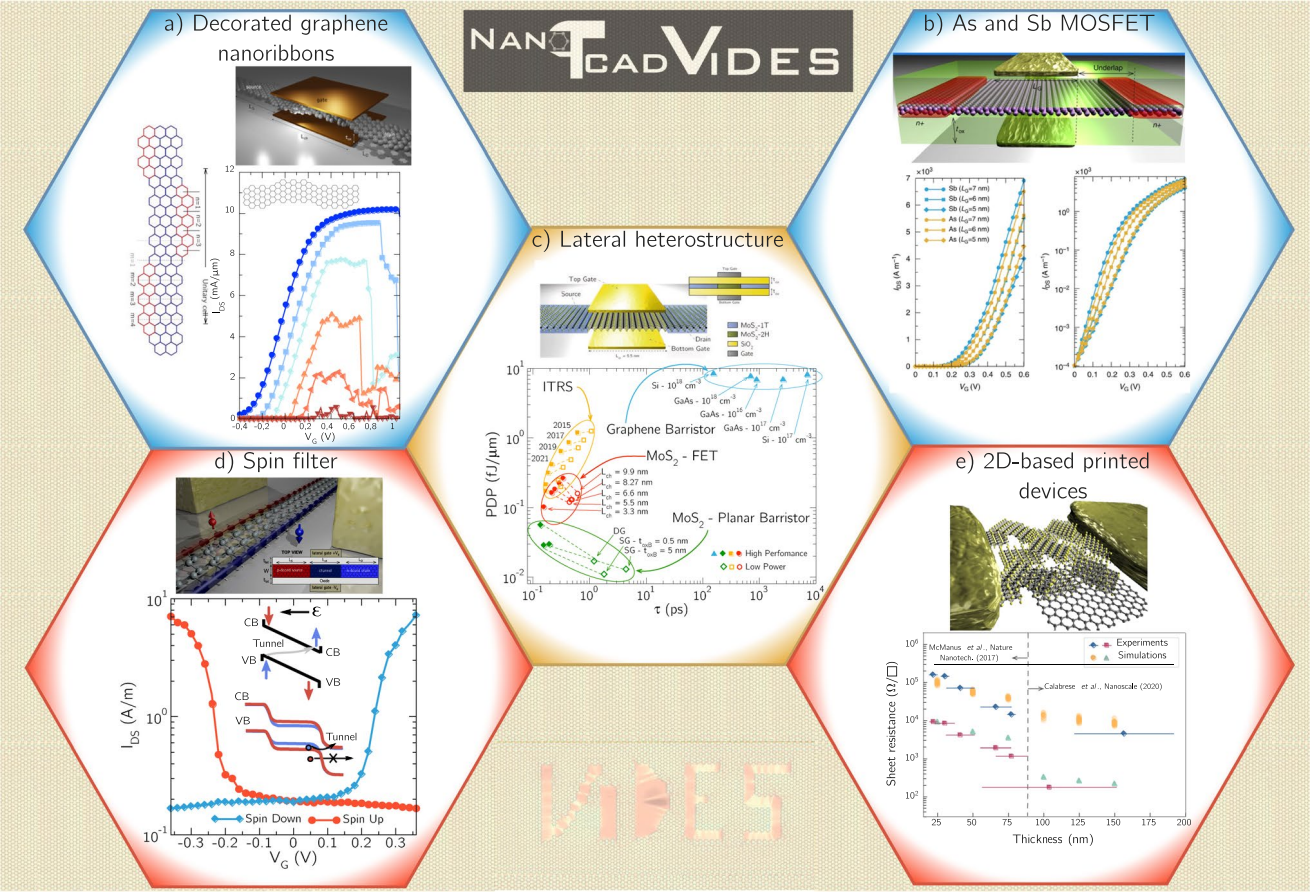
Examples of the use of NanoTCAD ViDES code: **a** decorated graphene nanoribbon with negative transconductance from Ref. [16], **b** arsenene and antimonene MOSFETs from Ref. [14], **c** lateral heterostructures made of different phases of MoS$_2$ from Ref. [9], **d** a spin-filter device based on monolayer stanene from Ref. [17] and **e** 2DM-based printed devices from Ref. [13]

We here collect some devices simulated with the NanoTCAD ViDES code. Figure 5 visually summarizes the presented devices: (a) decorated graphene nanoribbons with negative transconductance, (b) arsenene and antimonene MOSFETs, (c) lateral heterostructures made of different phases of MoS$_2$, (d) a spin filter device based on monolayer stanene and (e) 2DM-based printed devices. In the following, we briefly discuss each of them.

**Decorated graphene nanoribbons**.

Graphene nanoribbons (GNRs) have recently attracted renewed interest due to the possibility of engineering their crystal structure at the atom level [32]. In particular, the electronic properties of the GNR can be tuned by the proper arrangement of atoms, enabling the possibility of devising alternative devices based on carrier transport through topological states. In Fig. 5a, we report a few calculations realized employing NanoTCAD ViDES (from a recent work [16]) where, using a first-neighbor TB Hamiltonian based on p$_z$ orbital (very similar to the one in Eq. (2)), field-effect transistors (FETs) based on topological GNRs with shaped edges have been investigated. The theoretical calculations demonstrated the possibility of obtaining large negative differential transconductance effects as well as beating the Boltzmann limit for thermionic injection.

**Arsenene and antimonene FETs**.

While for graphene-based materials reliable simple first-neighbor tight-binding Hamiltonian can be used to simulate the electronic properties, the same does not apply to other, less known 2DMs, where a multiscale approach, as the one described in Sect. 3.2 is more appropriate. In Fig. 5b, we report the simulation realized using the code for 2D arsenene and antimonene as potential channel materials towards high-performance ultra-scaled FETs. The top panel shows a schematic illustration of As and Sb double gate FETs while in the bottom panel, the transfer characteristics for different channel lengths are shown demonstrating that ultra-scaled sub-10 nm devices based on 2D arsenene and antimonene comply with industry requirements [14].

**Lateral heterostructure of MoS**$_2$ **1T and 2 H phases**.

A step further in the multiscale approach, is the possibility to create either lateral or vertical heterostructures of 2DMs for future electronic devices as described in Sect. 3.2. In Fig. 5c, we recall the application of NanoTCAD ViDES for the study of one of such heterostructures, combining semiconductor and metal phases of MoS$_2$. This design was inspired by what experimentally has become a sort of quantum engineering of 2DMs enabled by the seamless combination of layers with different electronic properties, and, in particular, the demonstration of the possibility to obtain such heterostructures by electron beam irradiation [33]. In more detail, the code was used to investigate the behavior of a transistor (top panel) exploiting a lateral heterostructures of monolayer MoS$_2$, composed of adjacent regions of 1T (metallic) and 2 H (semiconducting) phases. The resulting figures of merit of the simulated devices, exhibited potential with respect to the foreseen evolution of CMOS technology, both for high-performance and low-power applications, as reported in [9].

**Spin filter based on 2D stanene**.

Beyond electronic transport, 2DMs offer new possibilities also in the field of spintronics, i.e. the transport and processing of the electron spin degree of freedom. NanoTCAD ViDES can simulate, by means of a multiscale approach, the spin transport features. In Fig. 5d, we collect its use to propose a novel device concept (top panel), based on a monolayer stanene nanoribbon, which exploited the presence of spin-polarized edge states in the nanoribbon, that could be tuned by applying an in-plane electric field. The calculations realized with the code demonstrated (bottom panel) spin-polarized currents up to a 98% with voltage-controlled spin polarization operating at room temperature and with small operating voltage (0.3 V). This device could be useful to explore new concepts of spin injectors or filters, that are fundamental building blocks of spintronics [17].

**2DM-based printed FETs**.

Printed electronics are one of the biggest opportunities for exploiting 2DMs, where inks prepared with various 2DMs flakes dispersions can be deposited layer-by-layer on a wide range of substrates to define different components in electronic circuits. We exemplify the use of the mesoscopic dissipative transport module of NanoTCAD ViDES applied to the study of these 2DM-based printed devices, as a low-cost, low-impact technological alternative. In Fig. 5e we bring back the study of 2D-based MoS$_2$- and graphene-printed FETs (top panels). The impact of the structure density and the variability on the mobility and sheet resistance was assessed and validated against experimental electrical measurements on printed graphene conductive lines as a function of film thickness (bottom panel) [34], demonstrating the strong potential of this module of the code as a guide for future experimental activity in the field [13].

## Conclusion

NanoTCAD ViDES software operation and main features are revised and updated. The two main modules of the code, i.e, the quantum elastic transport module, and the semi-classical dissipative transport module, are discussed in detail, presenting the code new functionalities, which include: (i) interfacing with Wannier90 and therefore with DFT suites for electronic structure calculations, that enables a fully multiscale approach for the study of nanometric devices, (ii) the inclusion of heterostructures of different 2DMs, allowing the simulation of devices based on lateral and van-der-Waals heterostructures, (iii) the modeling of mesoscopic layered structures, and 2DM-based printed devices as a paradigmatic case. Several examples of the application of the code are discussed to illustrate its remanent and up-to-date potential. The code additional capabilities and features are intended to maintain and broaden its appeal to the community, extending its range of applicability to new device designs, architectures, materials, and regimes of operation.

## Data Availability

Data will be made available upon reasonable request.

## Code Availability

The NanoTCAD ViDES software is available at the web page *http://vides.nanotcad.com*.
